# Multiparametric Immune Profiles and Their Potential Role in HIV-1 Disease Progression and Treatment

**DOI:** 10.3390/pathogens14040347

**Published:** 2025-04-04

**Authors:** Junwei Su, Junjie Zhang, Qianying Wang, Xiaojing Liu, Shuo Wang, Yuhua Ruan, Dan Li

**Affiliations:** 1 The Department of Infectious Diseases, State Key Laboratory for the Diagnosis and Treatment of Infectious Diseases, Collaborative Innovation Center for the Diagnosis and Treatment of Infectious Diseases, National Clinical Research Center for Infectious Diseases, The First Affiliated Hospital, School of Medicine, Zhejiang University, Hangzhou 310003, China; zjusujunwei@zju.edu.cn; 2 National Key Laboratory of Intelligent Tracking and Forecasting for Infectious Diseases, National Center for AIDS/STD Control and Prevention, Chinese Center for Disease Control and Prevention, Beijing 102206, China; zhangjj0673@163.com (J.Z.); wangqianyingchn@foxmail.com (Q.W.); xiaojing__liu@163.com (X.L.); wswangshuo1029@126.com (S.W.); ruanyuhua92@chinaaids.cn (Y.R.)

**Keywords:** HIV-1, immune profiles, disease progression, HAART, immune reconstitution

## Abstract

**Backgrounds:** The rapid initiation of highly active anti-retroviral therapy (HAART) can control HIV-1 viremia and stabilize the long-term health of people living with HIV-1 (PLWH). Despite this, individuals who are diagnosed late and exhibit poor therapeutic efficacy still pose a great challenge to global HIV management. To address this, we conducted comprehensive multiparametric immune profiling and analyzed its association with disease progression and therapeutic efficacy. **Methods:** Multicolor flow cytometry was used to characterize the circulating immune cell composition and cellular phenotypes in 40 treatment-naive individuals (16 chronic, 24 newly diagnosed), 26 HAART-treated individuals, and 18 healthy controls. Comparative analyses of T cell subsets, immune activation markers, and viral load signatures were performed, followed by network construction. We carried out principal component analysis and displayed the data by dimensionality reduction. **Results:** Persistent immune activation, dysregulated regulatory immunity, and aberrant memory differentiation markers were identified in T cells of HIV-1-infected individuals and were associated with disease progression. Additionally, HAART-treated patients which did not fully restore CD4 T cells exhibited higher levels of activated markers, suggesting possible biomarkers of therapeutic efficacy. **Conclusions:** This study describes changes in immune cell profiles throughout HIV-1 disease progression and explores suitable laboratory predictors for future clinical and therapeutic settings by monitoring pathological immune cell events.

## 1. Introduction

Although great achievements in prevention, diagnosis, care, and treatment strategies for HIV/AIDS control have been made, the disease remains a substantial health burden, particularly in low- and middle-income countries [1,2,3,4]. The main concerns include late diagnosis and poor therapeutic efficacy due to drug resistance and/or suboptimal adherence to HAART [5]. After the initial destruction of CD4+ T cells, pathological changes in the immune system during HIV-1 disease progression are driven by multiple inflammatory factors [6,7,8]. Continuous innovation in identifying biomarkers for monitoring disease progression and immune dysfunction remains critical, even among individuals receiving HAART [5,9].

Though both specific cellular and antibody immune responses contribute to HIV-1 acquisition control, viremia suppression, and the elimination of HIV-1-infected cells, only a small proportion of individuals can maintain undetectable plasma HIV-1 RNA levels and stable CD4+ T cell counts in the absence of antiviral therapy [10]. The “Test and Treat” strategy was implemented in China in 2016 to enhance epidemic control and improve health outcomes for people living with HIV-1. Although early ART initiation is established as effective in improving clinical outcomes, the persistence of the HIV reservoir requires lifelong antiretroviral therapy [3,4,11,12]. While HAART typically achieves virological suppression, treatment interruptions or drug resistance may lead to viral rebound and progressive immune deterioration [13,14,15,16,17,18].

During HIV-1 disease progression, dysregulated immune cell subsets orchestrate a complex interplay between viral persistence and host immunity: alteration in immune activation, memory T cells, regulatory T cells (Treg), and T helper 17 (Th17) cell populations exhibit both detrimental and protective effects [19,20]. Activated CD4^+^/CD8^+^ T cells, defined by CD38 and HLA-DR co-expression, serve as dual biomarkers for viral immune evasion and are clinically associated with disease progression [21,22,23]. Memory T cells are classified into central memory T cells (Tcm: CD45RA^-^CCR7^+^, which serve as long-term HIV-1 reservoirs due to their stem cell-like properties), effector memory T cells (Tem: CD45RA^-^CCR7^-^, dominant producers of IFN-γ), and effector T cells (Te: CD45RA^+^CCR7^-^), all of which mediate long-term immune responses against the HIV-1 virus. Among CD4+T cell subsets, Tregs and Th17 cells display opposing functions that regulate immune homeostasis in healthy individuals. Th17 cells maintain mucosal immunity by secreting IL-17/IL-22 to reinforce gut barrier integrity. Their early depletion triggers microbial translocation and systemic inflammation. Conversely, Tregs exacerbate viral persistence via CTLA-4-mediated dendritic cell suppression. The loss of Treg/Th17 balance critically drives HIV-1 pathogenesis and demands targeted investigation. Thus, comparative studies of treatment-naive patients at different stages of disease progression and HAART-treated participants with residual viremia may elucidate immune control mechanisms. Insights from natural HIV-1 infection could advance the identification of biomarkers for HIV-1 treatment and vaccine development.

In this study, we enrolled three cohorts, chronically HIV-1-infected patients with >10 years of untreated infection, newly HIV-1-infected patients (diagnosed within 1 year), and HIV-1 patients receiving HAART. We focused on dynamics changes in CD4+ and CD8+ T cell memory subsets, immune activation markers, and the balance between regulatory T cell subsets. By comparing these immune profiles, we aimed to elucidate the effects of HAART and the long-term consequences of untreated HIV-1 infection, thereby establishing a baseline to evaluate current treatment efficacy and guide strategies for comprehensive immune recovery and optimal patient outcomes.

## 2. Materials and Methods

### 2.1. Study Design and Participants

We enrolled three groups of HIV-1-infected individuals. The chronic infection group (CI) consisted of 16 HIV-1-infected patients who had not received HAART treatment for over ten years. The new infection group (NI) included 24 recently infected individuals who were infected within one year and remained untreated. The HAART-treated group (HAART-T) comprised 26 HIV-1-infected patients who maintained first-line regimens (2 NRTIs + 1 INSTI/NNRTI) for ≥5 years, though with suboptimal adherence (self-reported adherence < 80%) and treatment interruptions documented in most participants. A healthy control group (HC) of 18 adults, all negative for HIV-1, HBsAg, and anti-HCV antibodies, was also included. We analyzed gender, age, infection duration, peripheral blood CD4 and CD8 T cell counts, their ratios, and HIV-1 viral loads (VL) (Table 1). This study was approved by the Clinical Research Ethics Committee of the First Affiliated hospital, Collage of Medicine, Zhejiang University (20220077C), and informed consent was obtained from all participants.

### 2.2. CD4^+^ T Cell Count and HIV-1 Viral Load

CD4 T cell counts were quantified using BD TruCOUNT^TM^ tubes and flow cytometry with a set of antibodies, CD45 (PerCP), CD3 (FITC), CD4 (PE), and CD8 (APC), before analysis on a FACS Calibur. Plasma viral load detection employed the COBAS kit on a COBAS AMPLICOR PCR detector with a detection limit of 50 copies/mL.

### 2.3. Multicolor Flow Cytometry

Peripheral blood mononuclear cells (PBMCs) were thawed, rested overnight, and resuspended at 1 × 10^7^ cells/mL in RPMI-1640 + 10% FBS. For Th17 cell detection, PBMCs were incubated for 6 h, stained with surface antibodies (CD3, CD8, CD4), permeabilized, and stained intracellularly for IL-17A. Treg and memory T cell subsets were prepared by adjusting PBMCs to 1 × 10^7^ cells/mL, followed by surface staining with CD3, CD8, CD4, CD25, CCR7, CD127, and CD45RA and then permeabilization and intracellular staining with FoxP3. Immune activation status was assessed by surface staining PBMCs with CD3, CD45RA, HLA-DR, CD4, CD38, and CD27. Flow cytometry analysis for all samples was performed using FlowJo software (10.9). All the antibodies with detailed information were listed in Table S1.

### 2.4. HIV Reservoir Quantification

Total HIV-1 DNA quantification was conducted using a TaqMan-based PCR kit (supbio, Guangzhou, China) with specific primers and probes, ensuring accuracy through the inclusion of internal controls and nucleated cell controls. DNA was extracted from either whole blood or PBMCs and amplified using a dUTP/UDG system to minimize contamination risk. The reaction incorporated fluorescence detection, targeting HIV-1 DNA (FAM channel) and nucleated cells (VIC channel). Thermal cycling consisted of 40 cycles with fluorescence signals acquired during each cycle. Results were validated by ensuring the standard curve linearity met R^2^ ≥ 0.96. The assay demonstrated a detection limit of 20 copies per 10^6^ cells and a quantification range of 50 − 1 × 10^6^ copies per 10^6^ cells.

### 2.5. Statistical Analysis

Statistical analyses were performed using R-4.3.2. The heat map was generated with the pheatmap function from the ComplexHeatmap package (2.18.0) [24], and the data were scaled using the maximum–minimum standardization method to ensure consistency in visualization. A normality test was then performed to determine the data that exhibited a normal distribution. For data that met the normal distribution criteria, a comparison between two groups was performed using the *t*-test, a comparison between multiple groups was performed using analysis of variance (ANOVA), and a pairwise comparison between groups was performed using the Student–Newman–Keuls (SNK) method; for non-normally distributed data, a comparison between two groups was performed using the Wilcoxon rank-sum test (wilcox.test), a comparison between multiple groups was performed using the Kruskal–Wallis test (kruskal.test), and a pairwise comparison between groups was performed using the Wilcoxon rank-sum test. An image was drawn using the ggboxplot function of ggpubr. Effect sizes were quantified using Cohen’s d to assess the magnitude of the observed differences. Additionally, power calculations were conducted for the primary study endpoint. Principal component analysis (PCA) was drawn using the fviz_pca_biplot function of the factoextra package, and the data were displayed by dimensionality reduction. A correlation analysis was drawn using the corrplot function and the Spearman rank correlation test method. *p* < 0.05 was considered statistically significant.

## 3. Results

### 3.1. Increased Immune Activation Level During HIV-1 Infection

**Figure 1:**
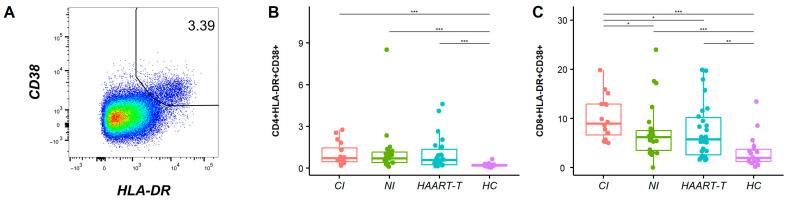
Comparison between immune activation groups. (**A**) The flow cytometry distribution diagrams of CD38*^+^* and HLA-DR*^+^* T cell subsets. Box plot comparing CD38 and HLA-DR expression levels of CD4*^+^* (**B**) and CD8*^+^* (**C**) T cells, CD4*^+^* and 8*^+^* T cell count, and VL in four groups: CI (red), NI (green), HAART-T (blue), and HC (purple). The Wilcoxon anecdotal test was used. *, *p* < 0.05; **, *p* < 0.01; ***, *p* < 0.001.

Multicolor flow cytometry was employed to quantify immune activation phenotypes across six T cell subpopulations during various stages of HIV-1 infection. The expression of CD38 and HLA-DR on T cells was used to identify the frequency of activated cells and was used for comparison in this study. As previously reported, the proportion of CD38 and HLA-DR co-expression better represents immune activation status (Figure S2D,E). The percentages of combined CD38*^+^*HLA-DR*^+^* subsets show distinct patterns in HIV-1-infected patients on CD4*^+^* and CD8*^+^* T cells, with the highest levels in chronically infected patients and then newly infected and HAART-treated patients (CD4*^+^*: CI: 1.06 ± 0.83%, *p* < 0.001, NI: 1.12 ± 1.66%, *p* < 0.001, HAART-T: 1.02 ± 1.17%, *p* < 0.001, all compared with HC, 0.22 ± 0.13%; CD8*^+^*: 10.01 ± 4.43%, *p* < 0.001, 7.30 ± 5.49%, *p* < 0.001, 7.44 ± 5.70%, *p* < 0.01, all compared with HC, 3.17 ± 3.29%) (Figure 1B,C and Figure S3A).

### 3.2. Altered Distribution of Memory T Cell Subsets in HIV-1-Infected Individuals

We performed a detailed phenotypic characterization of three memory T cell subsets defined by CD45RA and CCR7 expression: central memory T cells (Tcm: CD45RA*^-^*CCR7*^+^*), effector memory T cells (Tem: CD45RA*^-^*CCR7*^-^*), and effector T cells (Te: CD45RA*^+^*CCR7*^-^*), which mediate long-term immune responses.

**Figure 2:**
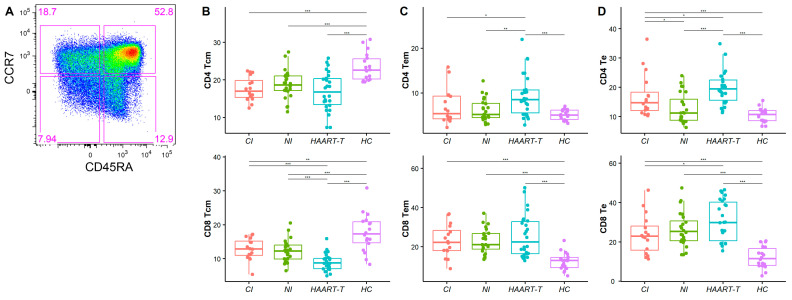
Comparison between memory T cell subsets. (**A**) The flow cytometry distribution diagrams of memory T cell subsets. Box plot comparing CD4 and CD8, memory T cell (Tcm) (**B**), effector memory T cell (Tem) (**C**), and effector T cell (Te) (**D**) levels in four groups: CI (red), NI (green), HAART-T (blue), and HC (purple). The Wilcoxon anecdotal test was used. *, *p* < 0.05; **, *p* < 0.01; ***, *p* < 0.001.

In HIV-infected patients, both CD4 and CD8 Tcm frequencies were significantly reduced compared to healthy controls (CD4 Tcm: CI: 17.56 ± 3.20%, *p* < 0.001; NI: 18.76 ± 3.81%, *p* < 0.001; HAART-t: 16.90 ± 5.07%, *p* < 0.001, all compared with HC: 23.42% ±3.63%; CD8 Tcm: CI: 12.85 ± 3.21%, *p* < 0.01; NI: 12.29 ± 3.30%, *p* < 0.001; HAART-t: 8.96 ± 2.51%, *p* < 0.001, all compared with HC: 17.67 ± 5.52%) (Figure 2B). This suggests a depletion of immune memory reserves in HIV-1-infected patients. While HAART has shown some efficacy in partially restoring CD4 Tcm levels, CD8 Tcm remained significantly lower even under treatment (8.96 ± 2.51% vs. HC, *p* < 0.001; vs. CI, *p* < 0.001; vs. NI, *p* < 0.001), indicating incomplete immune recovery.

Conversely, Tem and Te were expanded in HIV-1-infected patients, with peak frequencies observed in the HAART-treated group (CD4 Tem: 9.46 ± 4.72% vs. HC 5.11 ± 1.17%, *p* < 0.001, CD8 Tem: 25.58 ± 10.86% vs. HC 12.55 ± 4.48%, *p* < 0.001, CD4 Te: 20.03 ± 5.98% vs. HC 10.49 ± 2.42%, *p* < 0.001, CD8 Te: 30.74 ± 10.16% vs. HC 12.10 ± 5.46%, *p* < 0.001) (Figure 2C,D and Figure S3B). Furthermore, CD4 Tem and CD4 Te levels were significantly higher in HAART-treated patients compared to those with chronic infection (Tem: 6.95 ± 3.95% vs. HAART-t, *p* < 0.05; Te: 17.08 ± 7.42% vs. HAART-t, *p* < 0.05) and new infections (Tem: 6.14 ± 2.49% vs. HAART-t, *p* < 0.01; Te: 12.85 ± 5.14% vs. HAART-t, *p* < 0.001) (Figure 2C,D), implying sustained immune activation despite therapy.

### 3.3. Treg and Th17 Cell Analysis Revealed Incomplete Immune Recovery in HIV-Infected Patients

**Figure 3:**
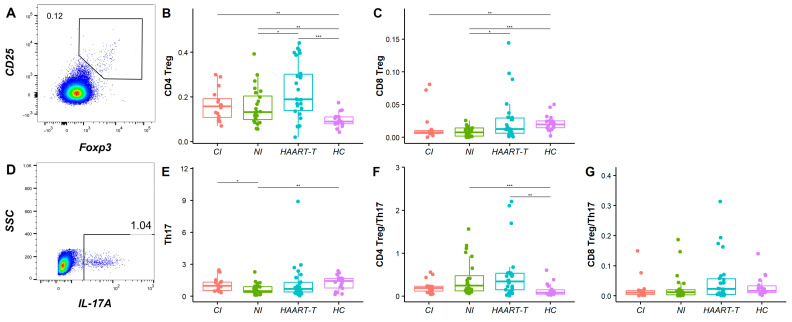
Comparison of regulatory cell subpopulation levels between HIV-infected patients and healthy controls in different groups. (**A**) The flow cytometry distribution diagrams of Treg subset. Box plot and flow chart comparing CD4 Treg (**B**), CD8 Treg (**C**), Th17 (**E**), and CD4 and CD8 Treg/Th17 (**F**,**G**) levels in four groups: CI (red), NI (green), HAART-T (blue), and HC (purple). The Wilcoxon anecdotal test was used. *, *p* < 0.05; **, *p* < 0.01; ***, *p* < 0.001. (**D**) The flow cytometry distribution diagrams of the Th17 cell subset.

We analyzed regulatory T cells (Treg, CD25*^hi^*Foxp3*^+^*) and Th17 cells (CD4*^+^*IL-17*^+^*) to evaluate immune homeostasis across HIV-1-infected cohorts. In the HAART-treated group, CD4 Treg levels (0.23 ± 0.13%) were significantly higher than that in newly infected patients (0.16 ± 0.08% vs. HAART-t, *p* < 0.05), while healthy controls (0.10 ± 0.03% vs. HAART-t, *p* < 0.001) had the lowest level (Figure 3B). CD8 Tregs were lower in chronically infected (0.02 ± 0.02% vs. HC, *p* < 0.01) and newly infected patients (0.01 ± 0.01% vs. HC, *p* < 0.001) than in healthy controls (0.02 ± 0.01%), while HAART-treated patients (0.03 ± 0.03% vs. HC, *p* > 0.05) had similar levels to healthy controls (Figure 3C), suggesting that HAART may activate specific immune regulatory mechanisms (Figure S3C).

Th17 cells, essential for pathogen defense and mucosal integrity, differed significantly between newly infected patients (0.68 ± 0.50%) and both chronically infected (1.09 ± 0.63% vs. NI, *p* < 0.05) and healthy controls (1.27 ± 0.68% vs. NI, *p* < 0.01) (Figure 3E). The CD4 Treg/Th17 ratio was significantly higher in HAART-treated patients (0.49 ± 0.59% vs. HC, *p* < 0.01) and newly infected patients (0.42 ± 0.42% vs. HC, *p* < 0.001) compared to healthy controls (0.14 ± 0.15%), indicating immune imbalance. No significant difference was found in the chronically infected group (0.21 ± 0.16%, *p* > 0.05, *p* > 0.05, *p* > 0.05), suggesting that long-term HIV-1 infection may restore balance (Figure 3F). However, HAART does not fully re-establish this balance. Although no significant differences were noted in the CD8 Treg/Th17 ratio, the slightly higher ratio in HAART-treated patients may reflect the treatment’s impact on immune equilibrium (Figure 3G and Figure S3C).

### 3.4. Persistent Immune Activation in T Cell Profiles Underscores the Challenges of Achieving Optimal Immune Restoration and Viral Suppression in HIV Patients

**Figure 4:**
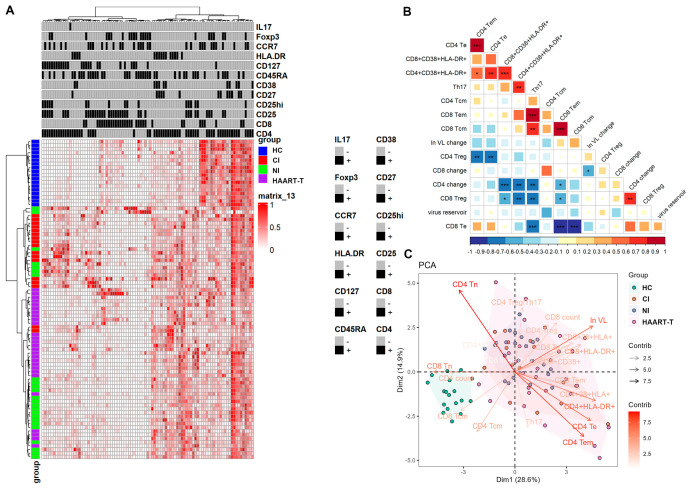
Correlation analysis between different cellular immune status and disease progression in HIV-infected patients. (**A**): Heat map analysis of major T cell subset immune molecules/cytokines related to the immune status of HIV-1-infected patients and normal controls. (**B**): Analysis of the correlation between different cellular immune status and disease progression in infected patients. Using Pearson correlation analysis, *, *p* < 0.05; **, *p* < 0.01; ***, *p* < 0.001. (**C**): Principal component analysis of each cell marker and each cell subpopulation in the four groups of healthy controls (red), chronically infected persons (green), newly infected persons (blue), and HAART-treated persons (purple).

We used multicolor flow cytometry to assess key immune molecules and cytokine profiles in T cell subsets, such as CD4, CD8, CD25, CD27, CD38, CD45RA, CD127, HLA-DR, CCR7, Foxp3, and IL-17, to evaluate post-HIV-1 infection immune dynamics. Hierarchical clustering stratified immune signatures between healthy controls (HC) and HIV-infected patients, who were further categorized into three subgroups based on their original classification (Figure 4A). Despite this classification, significant individual variability in marker expression was observed, indicating that HIV-1 disease progression and treatment are strongly associated with immune activation and regulation. For example, elevated CD38 and HLA-DR expression in chronically or newly infected patients indicate intense immune activation, while HAART treatment reduces these markers but does not normalize them to healthy levels (Figure 4A and Figure S3A). This suggests persistent immune activation despite on HAART, indicating possible suboptimal treatment efficacy.

Our correlation analysis revealed multidimensional interactions among viral load (VL), immune activation, and T cell subset dynamics in HIV-1 infection. Changes in CD4*^+^* T cell counts were significantly negatively correlated with the activation markers CD38 and HLA-DR, as well as with Th17 cell levels. Similarly, changes in CD8*^+^* T cell counts showed a negative correlation with VL levels. Additionally, we did find a negative correlation trend between the viral repository and most immune indicators, although this trend did not reach statistical significance (Figure 4B). A persistently high viral load exacerbates immune activation and CD4*^+^* T cell depletion while also driving compensatory regulatory mechanisms. In HAART-treated patients, CD4*^+^* T cell count negatively correlated with CD4 Tem and other activated subsets, while VL showed the opposite trend, suggesting that poor viral suppression exacerbates immune activation and CD4*^+^* T cell depletion (Figure S3A,B). Elevated CD4 Treg levels tracked with sustained VL, reflecting the dual pathology of chronic activation and counter-regulatory adaptation (Figure S3C).

Our principal component analysis (PCA) revealed stratified immune landscapes between healthy controls and HIV-infected individuals, with the latter characterized by persistent immune activation and reduced naive and central memory T cell subsets. Viral load emerged as a critical determinant of PCA clustering, underscoring its central role in disease progression and immune system dysregulation. The heterogeneity observed among HAART-treated patients underscores the complex interplay between viral load, treatment response, and immune reconstitution. These findings emphasize the need for personalized therapeutic strategies to address the diverse immune responses in HIV-1-infected individuals (Figure 4C and Figure S4).

### 3.5. Elevated Immune Activation and Persistent Viral Replication Contribute to Poor Immune Reconstitution in HAART-Treated HIV Patients with Low CD4 Counts

**Figure 5:**
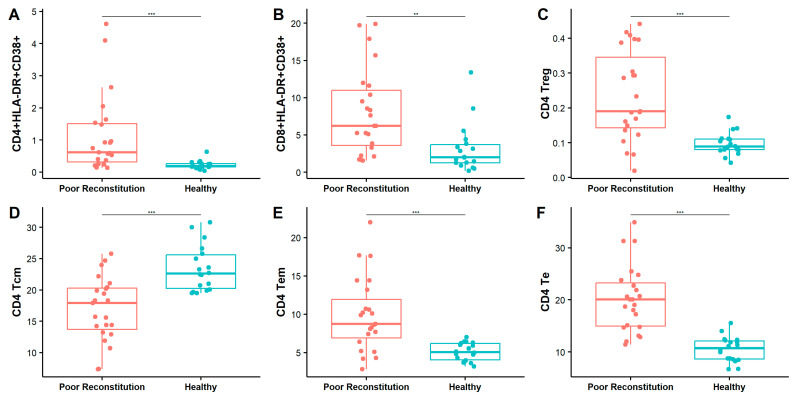
Comparison of immune activation status between poor immune reconstitution and healthy controls. Box plot comparing HLA-DR and CD38 co-expression levels on CD4 (**A**) and CD8 (**B**) T cells, CD4 Treg (**C**), CD4 Tcm (**D**), CD4 Tem (**E**), and CD4 Te (**F**) with poor immune reconstitution (red) versus HC (blue). The Wilcoxon anecdotal test was used. **, *p* < 0.01; ***, *p* < 0.001.

In our study, a CD4^+^ T cell count threshold of 500 cells/μL was used to stratify immune reconstitution outcomes in HAART-treated patients. Patients with a CD4 count above 500 were classified as having favorable immune reconstitution (*n* = 3), while those with a CD4 count below 500 were categorized as having poor immune reconstitution (n = 23). Notably, the co-expression levels of CD38 and HLA-DR were significantly elevated in patients with poor immune reconstitution compared to healthy controls, across both CD4 T cells (poor reconstitution: 1.12 ± 1.21% vs. HC: 0.22 ± 0.13%, *p* < 0.001) and CD8 T cells (poor reconstitution: 8.07 ± 5.77 vs. HC: 3.17 ± 3.29%, *p* < 0.01) (Figure 5A,B). The levels of CD4 Treg, CD4 Tem, and CD4 Te in the group with poor immune reconstitution were significantly higher than those in the healthy controls (CD4 Treg: poor reconstitution: 0.24 ± 0.13% vs. HC: 0.10 ± 0.03%, *p* < 0.001; CD4 Tem: poor reconstitution: 9.90 ± 4.81% vs. HC: 5.11 ± 1.17%, *p* < 0.001; CD4 Te: poor reconstitution: 20.20 ± 6.35% vs. HC: 10.49 ± 2.42%, *p* < 0.001), while the levels of CD4 Tcm were the opposite (CD4 Tcm: poor reconstitution: 16.95 ± 5.12% vs. HC: 23.42 ± 3.63%, *p* < 0.001) (Figure 5C–F and Figure S5C,D).

In addition, the CD4/CD8 ratio remained inverted, and the viral load was higher in patients with poor immune reconstitution (Figure S5A), indicating that HAART may not fully suppress viral replication in some cases. The elevated viral load and chronic inflammation likely drive the increased immune activation observed in these patients (Figure 5A,B and Figure S5B). Persistent immune activation can lead to T cell exhaustion, weakening the immune response and worsening the effects of HIV. These findings underscore the challenge of controlling ongoing immune activation and viral replication in some patients, potentially due to suboptimal adherence in antiviral therapy. No significant difference was observed between the poor reconstitution group and the immune reconstitution group, which may have been caused by the small sample size of the immune reconstitution group (Figure S6).

## 4. Discussion

While evidence demonstrates that clinical outcomes in HIV-1-infected individuals are intricately linked to host immune responses [25,26], our understanding of the mechanisms underlying the influence of late diagnosis and inadequate immune reconstitution is still limited and requires further characterization. To address this knowledge gap, we systematically analyzed immune responses post HIV-1 infection, focusing on memory, immune activation, and regulatory features across different disease stages, including individuals who were chronically and recently infected without HAART, those with a prolonged period of HAART, and healthy controls.

The expression of CD38 and HLA-DR serves as a validated biomarker of immune activation in HIV-1 pathogenesis. While effective HAART typically attenuates immune activation by suppressing viral replication, persistent activation remains a challenge in cases of suboptimal treatment. Consistent with the findings of Shete et al., our data indicate that HAART-treated patients, despite prolonged but suboptimal antiretroviral therapy (ART), maintain persistently lower CD4^+^ T cell counts and exhibit heightened T cell activation compared to individuals who achieve complete viral suppression [27]. This sustained T cell activation is a major driver of CD4^+^ T cell depletion, primarily attributed to ongoing low-level HIV-1 replication [28]. Furthermore, persistent immune activation is linked to impaired CD4^+^ T cell function [29,30], characterized by elevated expression of activation markers such as CD38 and HLA-DR [31,32], and contributes to the progression toward immune exhaustion. These immunological disturbances not only compromise immune memory but also disrupt the delicate balance between regulatory T cells (Treg) and Th17 cells, further complicating immune reconstitution and recovery [33]. Our study revealed significant heterogeneity in immune phenotypes among HIV-1-infected patients, particularly those with poor immune reconstitution. We found that the HAART-treated group exhibited immune profiles resembling those of newly infected individuals, suggesting that ineffective viral suppression may have sustained immune activation at levels comparable to early-stage infection. Moreover, the presence of elevated HLA-DR^+^CD38^+^ expression and evidence of chronic immune system damage further suggest an increased potential for viral escape from immune surveillance, particularly in the context of low CD4^+^ T cell counts. These findings underscore the need for more effective therapeutic strategies that target both viral suppression and immune system restoration to improve treatment outcomes in patients facing poor immune reconstitution. Suboptimal HAART disrupts T cell homeostasis, inducing chronic immune activation, naïve T cell exhaustion, and memory/effector subset imbalances. Despite HAART’s ability to reduce viral replication, persistent immune activation continues, contributing to CD4*^+^* T cell loss and immune exhaustion.

Prolonged HAART is closely associated with a marked reduction in the absolute counts of CD4^+^ central memory T cells (Tcm), which are essential for maintaining the T cell pool and generating effector cells [34,35,36]. These T cell subsets—including Tcm, effector memory T cells (Tem), and terminal effector T cells (Te)—play distinct yet interconnected roles in sustaining immune responses, establishing immunological memory, and controlling viral replication. Among these, Tcm cells are particularly critical for long-term immune stability and represent a major reservoir for latent HIV infection, posing significant challenges to viral eradication efforts [37]. HIV-1 infection profoundly disrupts the balance and abundance of these subsets, further complicating immune restoration [36,38]. While HAART effectively halts viral replication and aids partial immune recovery, our findings indicate that memory T cell subsets remain compromised, indicating ongoing immune exhaustion. Reduced Tcm cells limit the reserved capacity to control HIV-1 and other pathogens. Persistent activation of Tem and Te cells, driven by ongoing viral presence, exacerbates chronic inflammation and further impairs immune function, contributing to immune exhaustion. The insufficient replenishment of central memory cells leaves the immune system vulnerable to opportunistic infections [39].

Treg and Th17 cells play critical yet opposing roles in immune homeostasis, particularly in protecting mucosal barriers and modulating chronic immune activation. In HIV-1 infection, Treg cells suppress excessive immune responses to prevent host tissue damage but simultaneously inhibit antiviral immunity by constraining HIV-1-specific effector functions [40,41]. Similar to prior research, in patients with suboptimal HAART responses, elevated Treg frequencies mitigate systemic immune overactivation yet paradoxically sustain viral persistence [42,43,44,45,46]. Additionally, we also found chronic immune activation in HIV-1-infected patients could promote sustained Treg expansion and contribute to incomplete immune recovery and viral persistence [47]. Concurrently, the reduction in Th17 cells, particularly early in infection, compromises mucosal integrity, increases pathogen susceptibility, and drives immune activation, a key factor in HIV-1 progression [48]. In our study, participants in the HAART-treated group displayed elevated Treg frequencies alongside reduced Th17 cell counts, particularly in individuals with poor immune reconstitution. This imbalance was accompanied by heightened CD38^+^HLA-DR^+^ T cell activation and low CD4^+^ T cell counts, despite prolonged ART. Such profiles reflect the persistence of immune activation and incomplete immune recovery, underscoring the complex interplay between immune regulation, viral suppression, and immunopathology. These results highlight the importance of restoring Treg/Th17 balance as a potential therapeutic target for controlling inflammation and improving immune homeostasis. Addressing this imbalance may help reduce pathological inflammation, enhance immunosuppressive capacity, and ultimately improve immune reconstitution in patients with suboptimal ART outcomes.

In our study, chronic HIV-1 infection is characterized by persistent viral replication, CD4*^+^* T cell depletion, and increased immune activation, all of which worsen without treatment. Newly infected patients, often in the latent phase, exhibit lower immune activation. While HAART suppresses viral replication and reduces immune activation, it does not fully restore immune function, particularly in individuals with suboptimal responses. This is evident from the altered memory T cell balance, sustained immune activation, and disrupted Treg/Th17 ratio, leading to ongoing immune exhaustion and compromised mucosal defenses. Elevated HLA-DR and CD38 markers in HAART-treated patients, especially those with poor immune reconstitution, suggest an increased risk of viral escape and disease progression. Limited adherence to antiretroviral therapy was noted in some cases, which may have contributed to the observed treatment failure. Additionally, the possibility of antiretroviral drug resistance cannot be ruled out, although resistance testing was not performed in this study. Clinically, these findings underscore the need for more targeted interventions to mitigate persistent immune activation and improve immune balance in HAART-treated individuals. Strategies such as adjunctive therapies aimed at reducing inflammation, enhancing T cell homeostasis, or modulating Treg/Th17 dynamics may provide additional benefits in achieving optimal immune restoration. Furthermore, closer monitoring of immune activation markers like CD38 and HLA-DR in HAART-treated patients could help identify those at risk of immune dysfunction despite viral suppression, guiding personalized therapeutic strategies.

Our study has several limitations. Although our cohort included HIV-1-infected individuals at various stages, those with well-reconstituted immune systems were underrepresented, potentially impacting the statistical robustness and generalizability of our findings. While prior studies indicate that CD4^+^ T cell counts tend to recover steadily with prolonged ART, our HAART-treated group had persistently low CD4^+^ T cell counts despite being in therapy for over five years. This suggests that prolonged immune dysfunction may persist even in long-term ART patients, particularly in those with incomplete viral suppression or poor adherence. Additionally, other unmeasured factors may have contributed to the observed outcomes, suggesting the need for further investigation to fully elucidate the underlying mechanisms. The sample size may have limited our ability to detect subtle immunological differences and reduced the statistical power of some analyses. Future studies with expanded cohorts would help to further validate our observations. Furthermore, the observational design identifies associations but does not establish causality. As a cross-sectional study, we were unable to investigate the correlation between immune marker and cytokine changes with immune restoration, nor could we assess the underlying causal mechanisms. These aspects warrant further exploration in future studies.

## 5. Conclusions

These findings emphasize the importance of initiating HAART early to prevent chronic immune activation and T cell depletion. Moreover, maintaining HAART effectiveness is crucial to avoid persistent immune activation and further complications. Addressing these issues requires a comprehensive approach that goes beyond viral suppression, aiming to restore immune function, enhance antiviral memory, and improve immune surveillance—key factors for more effective treatments and progress toward an HIV-1 cure.

## Figures and Tables

**Table 1:** Characteristics of HIV-1-infected patients and healthy controls.

Basic Features	Chronic Infection (CI)	Newly Infection (NI)	HAART Treated (HAART-T)	Healthy Control (HC)
Sample cases	16	24	26	18
Gender: male/female	9/7	24/0	13/13	9/9
Age (years, IQR)	41 (38, 45)	27 (24, 30)	34 (29, 44)	29 (27, 34)
Time of infection (years)	19.3 ± 1.4	0.4 ± 0.2	6.3 ± 0.9	-
CD4*^+^* T cell count (cells/µL)	632 ± 174	379 ± 164	355 ± 146	715 ± 182
CD8*^+^* T cell count (cells/µL)	1010 ± 575	1082 ± 506	962 ± 354	473 ± 163
CD4*^+^*/CD8*^+^* T cell ratio	0.59 ± 0.24	0.39 ± 0.21	0.37 ± 0.22	1.52 ± 0.42
Viral load (HIV-1 RNA copies/mL)	15,800 ± 119,439	13,700 ± 60,492	7150 ± 28,371	-

## Data Availability

The data presented in this study are available on request from the corresponding author.

## Supplementary Materials

The following supporting information can be downloaded at: https://www.mdpi.com/article/10.3390/pathogens14040347/s1, Table S1. Flow Cytometry Antibodies table and Materials used in experiments; Figure S1. Sample flow cytometry gating strategy. Sample flow cytometry gating of CD38*^+^* and HLA-DR*^+^* (A), Treg cell (B), Th17 cell (C) and memory T cell (D); Figure S2. Comparison of cell markers, CD4*^+^* T cell count, CD8*^+^* T cell count and viral load between groups; Figure S3. Correlation analysis between cellular immune status and disease progression in each group; Figure S4. Principal component analysis Contribution of each component; Figure S5. Comparison of immune activation status, memory subpopulation and regulatory cell subpopulation levels between groups with poor immune reconstitution and healthy controls; Figure S6. Comparison of immune activation status, memory subpopulation and regulatory cell subpopulation levels between groups with poor immune reconstitution and immune reconstitution.
