# Supplementary material for: Multiparametric Immune Profiles and Their Potential Role in HIV-1 Disease Progression and Treatment
Source: Pathogens. 2025 Apr 4;14(4):347. doi: 10.3390/pathogens14040347 (PMC12030533; doi:10.3390/pathogens14040347)
Supplement: Supplementary file 1 [file pathogens-14-00347-s001.zip › pathogens-3515240-supplementary.pdf]

***Table S1**. Flow Cytometry Antibodies table and Materials used in experiments.*

REAGENT or RESOURCE	SOURCE	IDENTIFIER
**Flow Cytometry Antibodies**		
**Th17 cells subset staining panel**		
CD3-APC	BD Pharmingen, San Diego, CA, USA	340440
CD8-Percp	BD Pharmingen, San Diego, CA, USA	347314
CD4-PE	BD Pharmingen, San Diego, CA, USA	347327
IL-17A-FITC	eBioscience, San Diego, CA, USA,	11-7179-73
**Treg cell and memory T cell subset staining panel**		
CD25-APC	BD Pharmingen, San Diego, CA, USA	340939
CCR7-PE-Cy7	BD Pharmingen, San Diego, CA, USA	557648
Foxp3-FITC	eBioscience, San Diego, CA, USA	11-4776-73
aRat IgG2a, κ-FITC	eBioscience, San Diego, CA,USA	11-4321
CD127-Pacific Blue	eBioscience San Diego, CA,USA	57-1278-73
CD45RA-Percp-Cy5.5	eBioscience, San Diego, CA,USA	45-0458-73
CD3-ECD	Beckman Coulter, Miami, FL,USA	PN IM2705U

CD8-APC Cy7	BD Pharmingen, San Diego, CA, USA	557834
CD4-PE	BD Pharmingen, San Diego, CA, USA	555347
**Immune activation status staining panel**		
CD27-APCCy7	BD Pharmingen, San Diego, CA, USA	341025
CD4-PECy7	BD Pharmingen, San Diego, CA, USA	557852
CD38-Percp-Cy5.5	BD Pharmingen, San Diego, CA, USA	551400
HLA-DR-FITC	BD Pharmingen, San Diego, CA, USA	555811
CD45RA-APC	BD Pharmingen, San Diego, CA, USA	550855
**Experimental supplies**		
Ionomycin	Sigma, St. Louis, MO,USA	I0634
Brefeldin A	Sigma, St. Louis, MO,USA	B7651
PMA	Sigma St. Louis, MO,USA	P8139
Fix-Perm Solution A	CALTAG, Burlingame, CA, USA	GAS001S-100
Fix-Perm Solution B	CALTAG, Burlingame, CA, USA	GAS002S-100
**Equipment**		
Class II biological safety cabinet	NuAire, Plymouth, MN, USA	N/A
FACS LSR Fortessa flow cytometer	BD, San Jose, CA, USA	N/A
FACS Calibur flow cytometer	BD, San Jose, CA, USA	N/A
COBAS AMPLICOR analyzer	Roche, Basel, Switzerland	N/A

1-15K high-speed centrifuge	Sigma, St. Louis, MO,USA	N/A
MEK-6108K white blood cell differential counter	NIHON KOHDEN, Tokyo, Japan	N/A
15K high-speed refrigerated centrifuge	Sigma, St. Louis, MO,USA	N/A
IKA-MS1/MS2 vortex shaker	IKA, Staufen, Germany	N/A
Pipette	20μL, 200μL, 1000μL, Eppendorf, Hamburg, Germany	N/A
Micropipette	2.5μL, Gilson, Villiers-le-Bel, France	N/A
Electronic pipette	Eppendorf, Hamburg, Germany	N/A
Digital temperature controlled water bath	PolyScience, Niles, IL, USA	N/A
**Disposable experimental supplies**		
EDTA anticoagulant vacuum blood collection tube	BD, San Jose, CA, USA	N/A
Centrifuge tube 15mL, 50mL	Corning Company, Corning, NY, USA	N/A
Pipette tip 20μL, 200μL, 1000μL	Corning Company, Corning, NY, USA	N/A
Pipette 1mL, 5mL, 10mL, 25mL	Corning Company, Corning, NY, USA	N/A
Sampling tank	Corning Company, Corning, NY, USA	N/A
Cell cryopreservation tube 1.8mL	Corning Company, Corning, NY, USA	N/A
Plasma tube 1.5mL	Corning Company, Corning, NY, USA	N/A
Eppendorf tube 1.5mL	Corning Company, Corning, NY, USA	N/A

Sterile flow cytometry sample tube	BD, San Jose, CA, USA	N/A
**Cell isolation and culture reagents**		
R10: Contains RPMI 1640, 10% fetal bovine serum (heat inactivated at 56°C for 30 minutes), penicillin (100U/ml), streptomycin (200ug/ml), 1% glutamine (1%), L-glutamine Aminoamide, store at 4°C for use within 1 week after preparation		
RPMI 1640	Hyclone Company, Logan, UT, USA	SH30022.01
Fetal bovine serum	Hyclone, Logan, UT, USA	SH30406.02
Hepes buffer	Sigma, St. Louis, MO,USA	H0887
Ficoll-paque TM Plus	Amersham Biosciences, USA	17-1440-03
PBS	Hyclone, Logan, UT, USA	SH30256.01
DMSO	Sigma, St. Louis, MO,USA	D2650
**CD4 + T cell absolute count reagent**		
CD3 FITC/CD8 PE/ CD45PerCP/CD4 APC, with TruCOUNT absolute counting tube	BD, San Jose, CA, USA	340491
CD45-PerCP		clone 2D1 (HLe-1)
CD3-FITC		clone SK7
CD4-APC		clone SK3
CD8-PE		clone SK1
FACS Lysing Solution (10×)	BD Biosciences, San Jose, CA, USA	349202
**Viral load test kit**		
Cobas Amplicor HIV-1 Monitor Kit	Roche, USA	

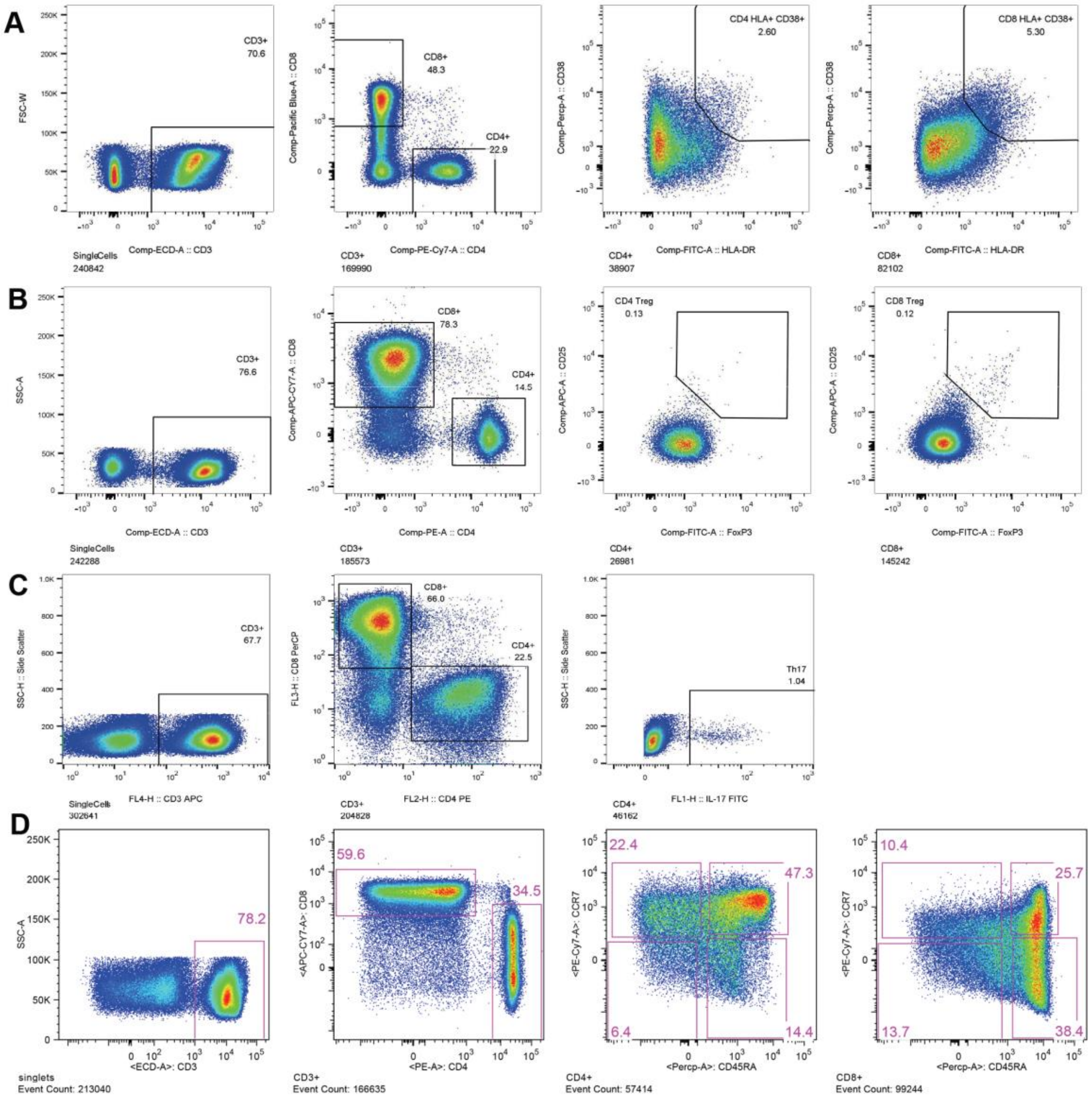

Figure S1. Sample flow cytometry gating strategy. Sample flow cytometry gating of $CD38^{+}$ and $HLA\text{-}DR^{+}$ (A), Treg cell (B), Th17 cell (C) and memory T cell (D).

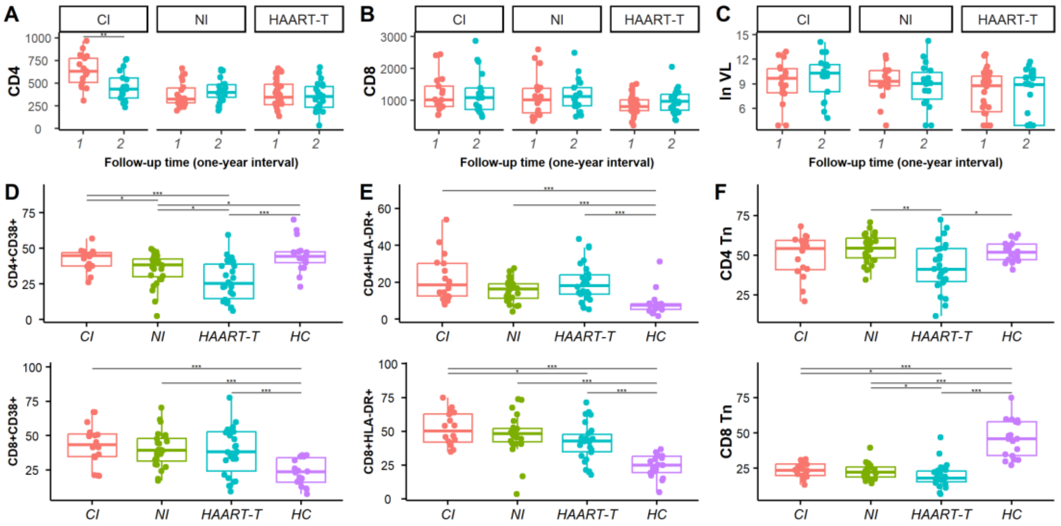

Figure S2. Comparison of cell markers, CD4^{+} T cell count, CD8^{+} T cell count and viral load between groups. Box plot comparing the differences in CD4^{+} T cell (A), CD8^{+} T cell (B) and VL (C) levels at two follow-up points(first follow-up in red, second follow-up in blue) among CI, NI, and HAART-T groups .Box plot comparing CD38 (D) and HLA-DR (E) expression levels and naïve T cells (Tn) (F) in four groups: CI (red), NI (green), HAART-T (blue) and HC (purple). The Wilcoxon anecdotal test was used. *, $p < 0.05$; **, $p < 0.01$; ***, $p < 0.001$.

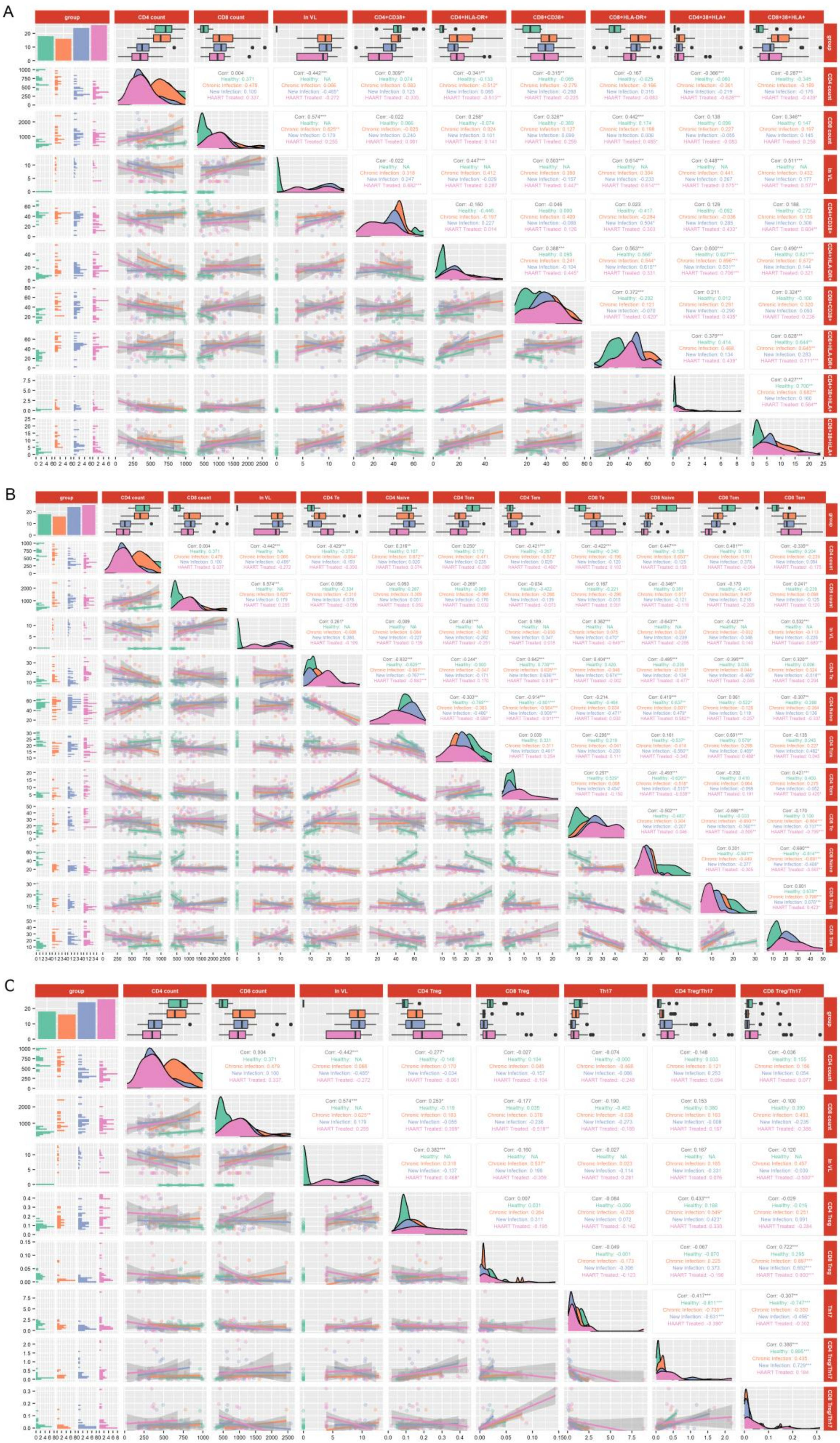
A
group
CD4 count
CD8 count
ln VL
CD4+CD38+
CD4+HLA-DR+
CD8+CD38+
CD8+HLA-DR+
CD4+38+HLA+
CD8+38+HLA+
B
group
CD4 count
CD8 count
ln VL
CD4 Te
CD4 Naive
CD4 Tcm
CD4 Tem
CD8 Te
CD8 Naive
CD8 Tcm
CD8 Tem
C
group
CD4 count
CD8 count
ln VL
CD4 Treg
CD8 Treg
Th17
CD4 Treg/Th17
CD8 Treg/Th17

Figure S3. Correlation analysis between cellular immune status and disease progression in each group. (A): Correlation analysis between immune activation status and disease progression within each group and between groups; (B): Correlation analysis between memory subgroups and disease progression within each group and between groups; (C): Adjustment within each group and between groups Correlation analysis of cell subpopulations and disease progression. Pearson correlation analysis was used. *, $p < 0.05$; **, $p < 0.01$; ***, $p < 0.001$.

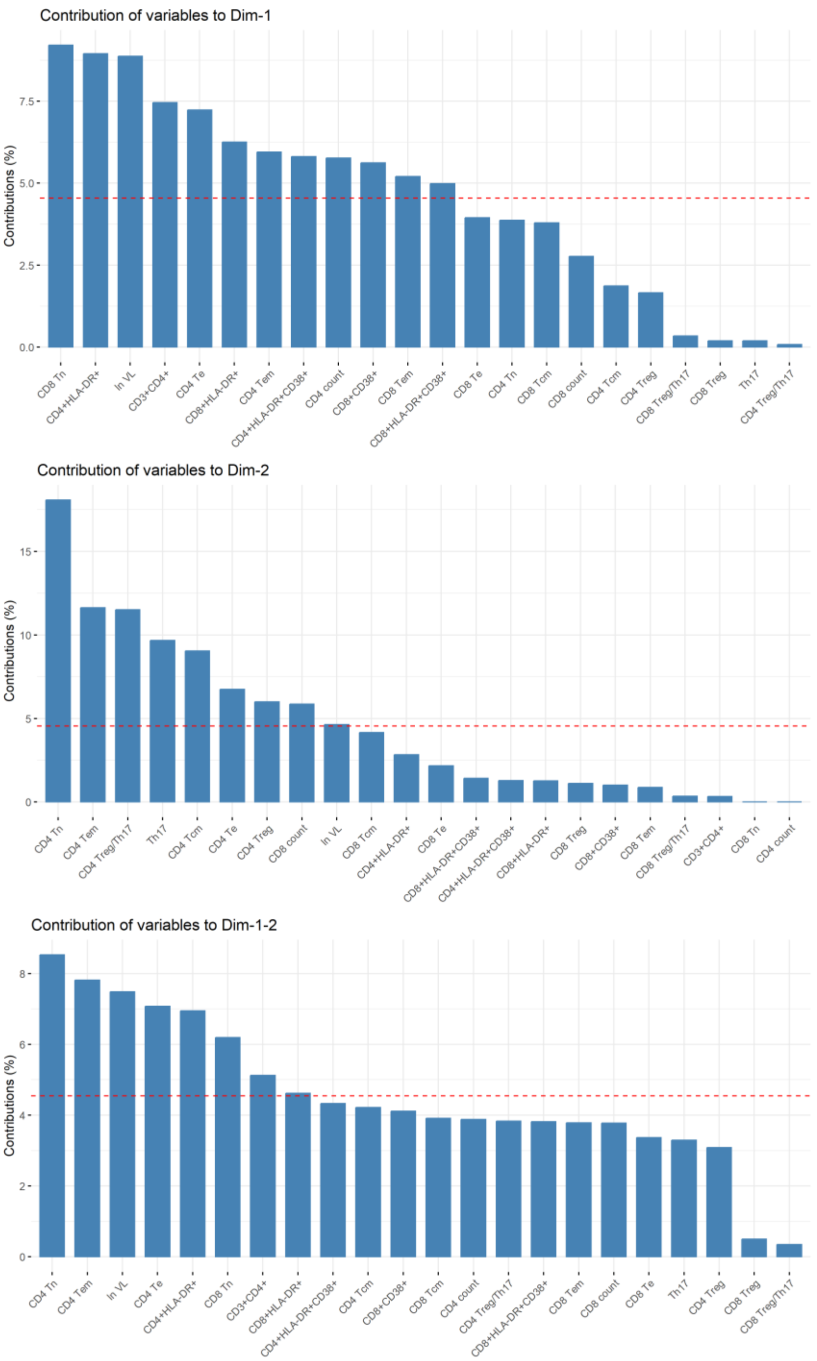

Figure S4. Principal component analysis Contribution of each component. The superimposed contribution rate of each variable in the first and second dimensions in PCA analysis.

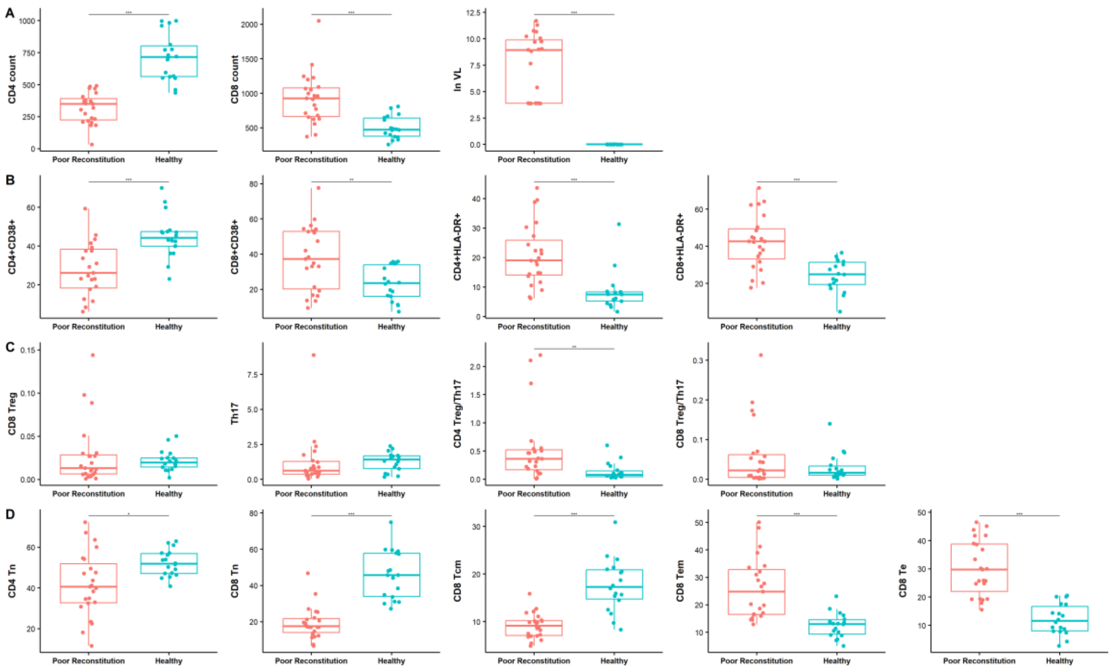

Figure S5. Comparison of immune activation status, memory subpopulation and regulatory cell subpopulation levels between groups with poor immune reconstitution and healthy controls. (A): Box plot comparing $CD4^+$ T cell & $CD8^+$ T cell count and natural logarithm of viral loads (ln VL) with poor immune reconstitution (red) versus healthy controls (blue). (B): Box plot comparing the CD38 and HLA-DR expression levels of CD4 and CD8 cells in the two groups. (C): Box plot comparing CD8 Treg, Th17, CD4 & CD8 Treg/Th17 in the two groups. (D): Box plot comparing CD4 and CD8 memory T cell levels in the two groups. The Wilcoxon anecdotal test was used. *, $p < 0.05$; **, $p < 0.01$; ***, $p < 0.001$.

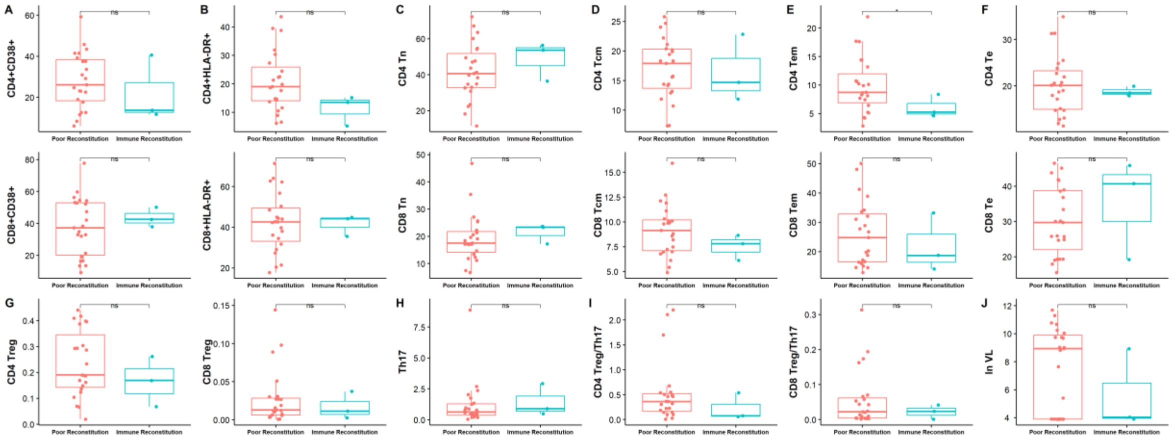

Figure S6. Comparison of immune activation status, memory subpopulation and regulatory cell subpopulation levels between groups with poor immune reconstitution and immune reconstitution. Box plot comparing CD38 (A), HLA-DR (B), Tn cell (C), Tcm cell (D), Tem cell (E), Te cell (F), Treg cell (G), Th17 (H), Treg/Th17 (I) and natural logarithm of viral loads (ln VL) level with poor immune reconstitution (red) versus immune reconstitution (blue). The Wilcoxon anecdotal test was used. *, $p < 0.05$; **, $p < 0.01$; ***, $p < 0.001$.
